# Editorial: The influence of internet and technology on mental health and psychological adjustment of young adults

**DOI:** 10.3389/fpsyt.2026.1866804

**Published:** 2026-05-12

**Authors:** Tao Xin

**Affiliations:** Beijing Normal University, Beijing, China

**Keywords:** concluding remarks, digital interventions, overview, protective factors, risk mechanisms

## An overview of the Research Topic

By providing a contemporary, multi−perspective snapshot of the field, this Research Topic makes several distinct contributions. The 19 articles presented here can be broadly clustered into three thematic strands—risk mechanisms, protective factors and positive applications, and digital interventions—each of which is summarized below.

## Risk mechanisms

One prominent strand of research within this Research Topic focuses on unpacking the mechanisms that underlie the relationship between technology use and poor mental health outcomes. Le Blanc−Brillon et al. conducted a comprehensive investigation into the links between social comparison on social media and young adults’ mental health, providing critical new insights into how upward social comparisons—a ubiquitous feature of platforms like Instagram and TikTok—drive psychological distress. Extending this risk−focused lens to non−social media contexts, Zhang et al. mapped the joint developmental trajectories of depressive symptoms and perceived stress across adolescence and examined how these co−developing patterns relate to smartphone addiction. Zhao et al. employed a rigorous cross−lagged panel model design to examine the transactional relationships between harsh parenting, shyness, and cyber victimization, revealing bidirectional pathways that underscore the need for family−level interventions. Yao et al. examined the psychological processes that bridge the gap between online verbal aggression and interpersonal trust among college students, showing how core self−evaluation and emotional intelligence operate as sequential mediators in this pathway. Şimşek and Başaran applied a manual three−step latent profile analysis to identify distinct phubbing (phone−snubbing) risk profiles among university students, moving beyond variable−centered approaches that treat phubbing as a unidimensional construct. Their findings highlight considerable heterogeneity in how young adults engage in phubbing behaviors, with important implications for targeted interventions. Finally, Kang et al. reported on a pilot study of a behavioral activation mobile application for depression among Korean young adults, providing encouraging evidence of the feasibility and promise of mobile−based interventions for this population. This work both identifies significant risks and illuminates practical pathways to mitigation.

## Protective factors, resilience, and positive applications of technology

A second, more hopeful strand of research shifts focus toward protective factors, individual resilience, and the positive applications of technology. In a pioneering study that challenges the prevailing deficit−focused narrative, Li et al. investigated whether single video games actually improve cognitive functioning in college students, employing both behavioral assessments and functional near−infrared spectroscopy (fNIRS) measures. Their findings provide compelling neuroscientific evidence that certain video games, under specific conditions, can enhance cognitive flexibility and executive function, opening new avenues for understanding technology as a tool for human development rather than simply as a risk factor. Meanwhile, Fan et al. examined the nuanced effects of media multitasking during online learning, distinguishing between relevant and irrelevant multitasking—a refinement that moves the field beyond simplistic “multitasking is harmful” pronouncements. James et al., in an opinion piece featured in this Research Topic, raise provocative questions about whether hedonic consumption behaviors in digital spaces should be viewed through the lens of behavioral addiction. Their contribution underscores the fine line between intense but healthy engagement and pathological overuse, encouraging more careful diagnostic conceptualization. Vega−Muñoz et al. provided a regional evidence base from Andean South America, shaping the digital well−being agenda for young adults in understudied contexts. Together, these studies provide essential building blocks for developing balanced, evidence−based recommendations that acknowledge both the opportunities and the risks associated with technology use.

## Digital interventions

A third strand of research provides direct evidence on the efficacy of digital tools for preventing and treating mental health problems. In addition to the mobile application pilot study by Kang et al., Zhou et al. described a rigorous study protocol for an internet−based peer education intervention targeting postpartum depression, offering a framework for scalable, low−cost mental health support for young mothers. These intervention studies collectively demonstrate that digital technologies, when thoughtfully designed and rigorously evaluated, hold considerable promise for expanding access to mental healthcare and enabling early intervention.

## Concluding remarks

The articles presented in this Research Topic collectively provide a timely, nuanced, and methodologically rigorous overview of the complex interplay between internet technology use and psychological adjustment among young adults. Strengths of the Research Topic include its geographic diversity, its bridging of risk and resilience perspectives, its inclusion of neuroscientific and longitudinal designs, and its progression from basic mechanism research through to applied intervention studies.

Nevertheless, the Research Topic also highlights several critical gaps and priorities for future inquiry. First, more longitudinal research is urgently needed to disentangle cause and effect in the relationships between digital technology engagement and mental health outcomes. While cross−sectional research can identify correlates and cross−lagged panel models can reveal temporal sequences—as exemplified by Zhang et al., Zhao et al., and others in this Research Topic—the field would greatly benefit from multi−wave prospective studies spanning the full transition from adolescence through to young adulthood. Such research could allow investigators to parse differential susceptibility and examine how early digital habits shape long−term developmental trajectories. Second, future research must move beyond aggregated screen−time metrics toward more nuanced, ecologically valid assessments of digital engagement. Greater attention should be directed to network analyses that can untangle the complex interrelationships among multiple digital behaviors and mental health symptoms simultaneously. Third, there is a pressing need to prioritize the co−design of digital interventions and prevention strategies with young adults themselves, ensuring that programs are relatable, acceptable, feasible, engaging, and genuinely effective in real−world settings. Fourth, researchers should further investigate person−environment interactions—for instance, how individual differences in temperament, emotion regulation capacity, or pre−existing vulnerabilities moderate young adults’ responses to digital environments—to enable truly personalized recommendations for healthy technology use. Fifth, cross−cultural and cross−national comparisons are essential for distinguishing universal mechanisms of digital engagement from culture−specific patterns, a need that this Research Topic’s geographic breadth has begun to address. Finally, given the ever−evolving digital landscape—from the rise of generative AI and immersive virtual reality to algorithm−driven content feeds—ongoing monitoring and rapid−cycle research will be necessary to ensure that evidence−based guidance keeps pace with technological change.

We hope that this Research Topic will serve as a valuable resource for researchers, clinicians, educators, parents, and policymakers who are committed to fostering healthy digital engagement and supporting the psychological well−being of young adults in the digital age. By synthesizing current evidence, identifying critical mechanisms and effective interventions, and charting a forward−looking agenda for future study, the contributions collected here advance the knowledge base needed to help young people navigate the digital world with resilience, balance, and well−being.

